# High Efficiency Drug Repurposing Design for New Antifungal Agents

**DOI:** 10.3390/mps2020031

**Published:** 2019-04-17

**Authors:** Jong H. Kim, Kathleen L. Chan, Luisa W. Cheng, Lisa A. Tell, Barbara A. Byrne, Kristin Clothier, Kirkwood M. Land

**Affiliations:** 1 Foodborne Toxin Detection and Prevention Research Unit, Western Regional Research Center, USDA-ARS, 800 Buchanan St., Albany, CA 94710, USA; kathy.chan@ars.usda.gov (K.L.C.); luisa.cheng@ars.usda.gov (L.W.C.); 2 Department of Medicine and Epidemiology, School of Veterinary Medicine, University of California at Davis, One Shields Avenue, Davis, CA 95616, USA; latell@ucdavis.edu; 3 Department of Pathology, Microbiology, and Immunology, School of Veterinary Medicine, University of California at Davis, One Shields Avenue, Davis, CA 95616, USA; bbyrne@ucdavis.edu (B.A.B.); kaclothier@ucdavis.edu (K.C.); 4 California Animal Health and Food Safety Laboratory, University of California at Davis, One Shields Avenue, Davis, CA 95616, USA; 5 Department of Biological Sciences, University of the Pacific, 3601 Pacific Avenue, Stockton, CA 95211, USA; kland@pacific.edu

**Keywords:** antifungal intervention, antioxidant system, *Aspergillus*, chemosensitization, drug repurposing, drug resistance, mutants, pathogen control

## Abstract

Current antifungal interventions have often limited efficiency in treating fungal pathogens, particularly those resistant to commercial drugs or fungicides. Antifungal drug repurposing is an alternative intervention strategy, whereby new utility of various marketed, non-antifungal drugs could be repositioned as novel antifungal agents. In this study, we investigated “chemosensitization” as a method to improve the efficiency of antifungal drug repurposing, wherein combined application of a second compound (viz., chemosensitizer) with a conventional, non-antifungal drug could greatly enhance the antifungal activity of the co-applied drug. Redox-active natural compounds or structural derivatives, such as thymol (2-isopropyl-5-methylphenol), 4-isopropyl-3-methylphenol, or 3,5-dimethoxybenzaldehyde, could serve as potent chemosensitizers to enhance antifungal activity of the repurposed drug bithionol. Of note, inclusion of fungal mutants, such as antioxidant mutants, could also facilitate drug repurposing efficiency, which is reflected in the enhancement of antifungal efficacy of bithionol. Bithionol overcame antifungal (viz., fludioxonil) tolerance of the antioxidant mutants of the human/animal pathogen *Aspergillus fumigatus*. Altogether, our strategy can lead to the development of a high efficiency drug repurposing design, which enhances the susceptibility of pathogens to drugs, reduces time and costs for new antifungal development, and abates drug or fungicide resistance.

## 1. Introduction

There have been continuous efforts to develop new antifungal agents or to improve the efficacy of conventional antifungal methods [1,2]. However, current intervention strategies often have limited efficiency in treating fungi, especially those pathogens resistant to drugs or fungicides [3]. The use of high-throughput screenings/bioassays to develop new antifungal agents and/or define cellular targets of newly-identified antifungal agents is still a developing field. This is especially true with regard to determining the involvement of specific genes, genetic pathways or previously undetected lipid changes in cellular membranes, cross talks between lipid molecules and mitochondrial dysfunction, cell wall integrity and filamentous fungal growth, etc., which can explain resistance to conventional antifungal agents [4,5,6,7].

Recently, increased incidences of fungal resistance to a class of azoles make fungal infections a global human health issue [8]. Aspergillosis is an example. Aspergillosis is a fungal disease caused by filamentous fungal pathogens in the genus *Aspergillus* [9]. Immuno-compromised groups of people or patients with lung diseases are especially at risk of developing aspergillosis. Among the several types of aspergillosis documented (such as allergic bronchopulmonary aspergillosis, allergic *Aspergillus* sinusitis, aspergilloma, chronic pulmonary aspergillosis, invasive aspergillosis (IA), and cutaneous aspergillosis) [10], IA is a particularly devastating infection triggered by environmental *Aspergillus* species, wherein *Aspergillus fumigatus* is the leading agent of IA followed by *A. flavus*, *A. terreus*, *A. niger*, and *A. nidulans* [9,10].

Certain azole fungicides, such as propiconazole or tebuconazole, that are applied to agricultural fields have the same mode of antifungal action as clinical azole drugs. Such long-term application of azole fungicides to fields could provide selection pressure for the emergence of pan-azole-resistant strains, such as the *A. fumigatus* TR34/L98H mutant [11,12]. As a result, there is a continuous need to improve the efficacy of current antifungal drugs or develop new intervention strategies. Of note, an invasive *A. fumigatus* infection (pulmonary) could also be acquired from contaminated foods, indicating IA further involves public food safety issue [13].

Considering the development of entirely new antifungal drugs is a capital-intensive and time-consuming process, an alternative approach termed “antifungal drug repurposing” has been recently investigated. Antifungal drug repurposing is the repositioning process of already marketed non-antifungal drugs—previously approved for treating other diseases—to control fungal infections [14]. One of the merits of drug repurposing is that the mechanisms of action, cellular targets or safety of the commercial drug has already been identified or characterized. However, although drug repurposing has become a viable approach to accelerate new antifungal drug development, this strategy still requires highly sensitive screening systems.

Meanwhile, antifungal “chemosensitization” has been developed as a new intervention method, where co-application of a second compound (viz., chemosensitizer; natural or synthetic), with a commercial drug has been found to enhance the antifungal efficacy of the co-applied drug [15]. The key advantage of chemosensitization is that, in contrast to combination therapy (viz., co-application of two or more commercial antifungal drugs), a chemosensitizer itself does not have to possess a high level of antifungal potency. Instead, a chemosensitizer causes the target pathogen to become more susceptible to the commercial co-applied drug by modulating the pathogen’s defense system to the drug. Chemosensitization could also overcome fungal resistance to certain commercial antifungal drugs [15].

In this proof of concept study, we tried to develop a high-efficiency drug repurposing method by targeting the fungal antioxidant system. We applied a previously developed chemosensitization strategy by including redox-active natural compounds or a structural analog as sensitizers, and also used fungal mutants lacking key genes in the antioxidant system. This resulted in the enhancement of the efficacy of the repurposed pro-oxidant drug bithionol. Results indicated that the sensitivity of the drug repurposing process could be augmented by the chemosensitization method and/or inclusion of fungal mutants lacking key genes in the cellular targets.

## 2. Materials and Methods

### 2.1. Literature Search: PubMed Database

Articles were retrieved via a PubMed search in the National Center for Biotechnology Information [16] (https://www.ncbi.nlm.nih.gov/) by using the key words “Drug Antifungal Repositioning” (Search date: May 31, 2018). The retrieved articles were re-evaluated further for the relevance of the contents to the subject antifungal drug development: Repositioning of non-antifungal drugs towards fungal control.

### 2.2. Chemicals

Chemical compounds, such as aspirin (acetyl salicylic acid), bithionol (2, 2’-sulfanediylbis (4, 6-dichlorophenol)), octyl gallate (octyl 3,4,5-trihydroxybenzoic acid; OG), thymol (2-isopropyl-5-methylphenol; THY), 4-isopropyl-3-methylphenol (4I3M), and 3,5-dimethoxybenzaldehyde (3,5-D), were procured from Sigma Co. (St. Louis, MO, USA). Each compound was dissolved in dimethylsulfoxide (DMSO; absolute DMSO amount: <2% in media) before incorporation into culture media. Throughout this study, controls (no treatment) contained DMSO at levels equivalent to that of cohorts receiving antifungal agents, within the same set of experiments.

### 2.3. Antifungal Bioassay

Antifungal activities of test compounds were examined in the wild type and two antioxidant mutants (*sakA*Δ, *mpkC*Δ) of the human pathogen *Aspergillus fumigatus* AF293 (see below for sources) and two mycotoxigenic fungi, *Aspergillus parasiticus* 2999 and *A. parasiticus* 5862 (National Center for Agricultural Utilization and Research, USDA-ARS, Peoria, IL, USA). Five μL of each compound was spotted onto the lawn of test fungi (1 × 10^4^ cfu/mL; potato dextrose agar (PDA) plates), and fungi were incubated at 35 °C for up to 48 hr. The formation of the zone of inhibition was monitored (with duplicates) at 24 and 48 hr of incubation.

### 2.4. Overcoming Fludioxonil Tolerance by Bithionol

Determination of overcoming fludioxonil tolerance of *A. fumigatus sakA*Δ and *mpkC*Δ mutants was based on comparison of fungal radial growth between treated and control colonies. Fungal conidia (5 × 10^3^) were diluted in phosphate buffered saline and inoculated as a drop onto the center of PDA plates (triplicates) containing: (1) No treatment (control); (2) Bithionol (125 μM); (3) Fludioxonil (50 μM); and (4) Bithionol + Fludioxonil. Growth was observed for 5 to 7 days at 35 °C.

### 2.5. Statistical Analysis

Statistical analysis (Student’s *t*-test) was performed based on Reference [17], where *p* < 0.05 was considered significant.

## 3. Results and Discussion

### 3.1. Aspirin and Bithionol

We initially performed a PubMed database search in the National Center for Biotechnology Information [16] (https://www.ncbi.nlm.nih.gov/) by using the key words “Drug Antifungal Repositioning” (Accessed on May 31, 2018), retrieving 70 articles. We re-evaluated the content of the retrieved articles for their relevance to antifungal drug development, identifying 16 articles (and references therein), which are shown in Table 1. The remaining 54 articles not selected described: (1) Antibacterial, antiviral, or antiprotozoal drug development; (2) anticancer drug development; and (3) drug development for other human diseases such as Parkinson’s disease, hematologic malignancy, etc. Pharmacological information of the repurposed compounds is also provided in the supplementary Table S1 [18,19,20,21,22,23,24,25,26,27,28,29,30,31,32,33,34,35,36,37,38].

**Figure 1:**
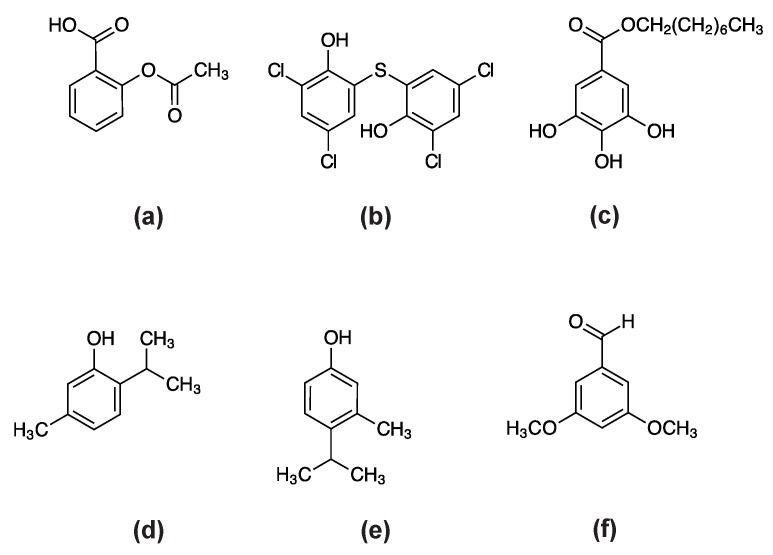
Structures of compounds examined in this study: (**a**) Aspirin; (**b**) bithionol; (**c**) octyl gallate; (**d**) thymol (2-isopropyl-5-methylphenol); (**e**) 4-isopropyl-3-methylphenol; (**f**) 3,5-dimethoxybenzaldehyde.

We chose “aspirin” and “bithionol” as representative redox-active drugs (for targeting the fungal antioxidant system) for further investigation (Figure 1a,b). Aspirin (acetyl salicylic acid) is a non-steroidal anti-inflammatory agent, while bithionol is a halogenated anti-protozoal drug. Both aspirin and bithionol have been known to participate in reactive oxygen species (ROS)-mediated apoptosis (programmed cell death) in cancer cells [54,55]. Octyl gallate (OG) was used as a positive control, which is a redox-active agent (possessing antioxidant and pro-oxidant activity) interrupting the lipid bilayer-protein interface in fungal cells (Figure 1c).

### 3.2. Chemosensitization to Enhance the Efficacy of Repurposed Drugs

Figure 1d,e show examples of chemosensitizers used in this study for targeting fungal antioxidant systems. Thymol (2-isopropyl-5-methylphenol) is a redox-active natural compound, and 4-isopropyl-3-methylphenol (4I3M) is a synthetic analog of thymol. We performed zone of inhibition bioassays, and compared the antifungal efficacy of repurposed drugs for wild type, antioxidant mutants, wild type with chemosensitizers, and antioxidant mutants with chemosensitizers.

Of note, the antioxidant systems of fungi, such as the mitogen-activated protein kinase (MAPK) signaling pathway, have been effective antifungal targets of redox-active agents. For instance, *A. fumigatus sakA*Δ and *mpkC*Δ are mutants lacking antioxidant MAPK genes [56,57]. Previous studies have shown that *A. fumigatus sakA*Δ and *mpkC*Δ mutants are highly susceptible to redox-active drugs such as amphotericin B or itraconazole compared to the wild type strain [58,59].

#### 3.2.1. Thymol as a Chemosensitizer to Bithionol or Aspirin

**Figure 2:**
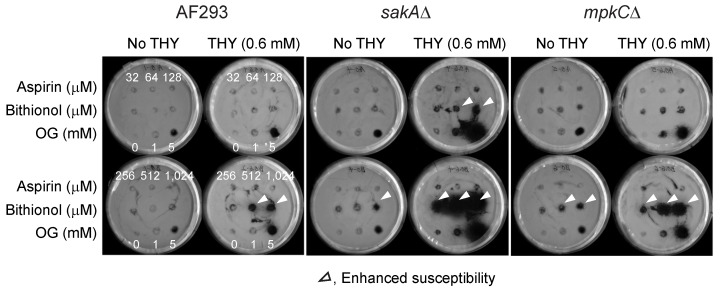
Enhancement of antifungal activity of bithionol by thymol tested in *A. fumigatus*. THY, thymol; OG, octyl gallate (positive control).

Results showed that antifungal activity of bithionol was greatly enhanced by thymol, while that of aspirin was almost not affected, indicating “drug-chemosensitizer specificity” exists for the enhancement of antifungal activity (Figure 2). Drugs were tested at 32 to 1024 μM, with or without 0.6 mM of thymol, and the positive control OG was tested at 1 and 5 mM. The results also showed that *A. fumigatus* MAPK mutants (*sakA*Δ, *mpkC*Δ) were more susceptible to the treatment compared to the wild type, indicating increased susceptibility of antioxidant mutants to the co-application of redox-active agents, such as thymol.

Bithionol is an anti-parasitic, “pro-oxidant” drug approved previously by the Food and Drug Administration [60]. It has recently been shown that co-application of bithionol sensitized ovarian cancer cells to paclitaxel, thus requiring lower doses of paclitaxel for cancer treatment [54]. Bithionol synergistically interacted with paclitaxel, where the combined application (bithionol + paclitaxel) increased the generation of ROS and also enhanced apoptosis in cancer cells [54]. In cancer therapy, generation of ROS is the key mechanism of apoptosis for chemotherapeutic reagents. Therefore, increased generation of cellular ROS via combined application of chemotherapeutic drugs with bithionol contributed to the enhancement of cancer therapy [54]. Co-application of bithionol could also mitigate the toxic side-effects associated with the high dose treatment of the drug paclitaxel [54]. A similar phenomenon was also observed with the anticancer drug cisplatin, where bithionol augmented the susceptibility of cisplatin-resistant cell lines to the drug by increasing ROS generation in the cells [61].

On the other hand, although aspirin has been shown to participate in ROS-mediated apoptosis in cancer cells, aspirin played a role as an “antioxidant” until the start of apoptosis in cells, thereby indicating ROS were not the major factors to trigger apoptosis in aspirin-treated cells [55].

Thymol is a redox-active agent, and therefore acts as an antioxidant at lower concentrations. At higher concentrations, it functions as a pro-oxidant, inducing oxidative stress in the Caco-2 cell line [62]. We speculate that, as observed in anticancer therapy, combined application of bithionol and the redox-active thymol could synergize to enhance cellular oxidative stress, which resulted in increased sensitivity of fungi (i.e., enhanced zone of inhibition, Figure 2) to the treatments, especially in mutants having defects in antioxidant systems. 

#### 3.2.2. 4-Isopropyl-3-Methylphenol as a Chemosensitizer to Bithionol or Aspirin

**Figure 3:**
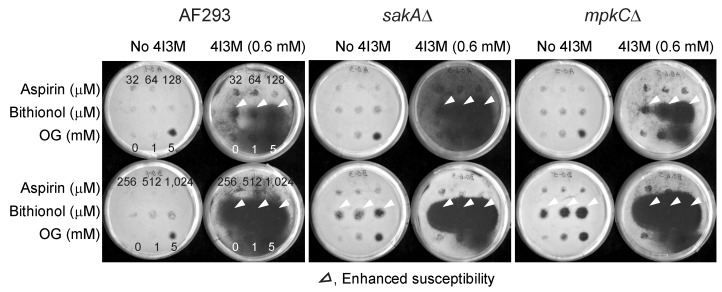
Enhancement of antifungal activity of bithionol by 4I3M tested in *A. fumigatus*. 4I3M, 4-isopropyl-3-methylphenol; OG, octyl gallate (positive control).

The level of bithionol activity was enhanced further when 4I3M, a structural analog of thymol, was co-applied as a chemosensitizer (Figure 3). For example, the zone of inhibition with 4I3M was detected at a much lower concentration of bithionol, namely 32 to 128 μM, while that with thymol was detected at 512 to 1024 μM of bithionol. Also, the sizes of zone of inhibition with 4I3M were larger than that with thymol. Therefore, results indicated that 4I3M could be more effective chemosensitizer to bithionol, when compared to thymol in *A. fumigatus* (Figure 3). As observed in thymol, the activity of aspirin was almost not affected by co-treatment with 4I3M.

Recent studies showed that the yeast *Saccharomyces cerevisiae* has served as a useful system for the identification of new antifungal agents and their cellular targets in view that: (1) The *S. cerevisiae* genome has been sequenced and well annotated [63], and (2) around 6,000 haploid gene deletion mutant collections of *S. cerevisiae* have been the tool for determining drug mode of action [64,65,66]. *Aspergillus* species and the model yeast *S. cerevisiae* also share high homology in the structure of their antioxidant MAPK signaling systems [67]. This means the genetic or genomic resources of *S. cerevisiae*, such as gene deletion mutants of the yeast, could serve as tools for drug screening for control of *Aspergillus* species [64,65,66].

Yeast dilution bioassay in a prior study showed the “sensitive” responses of yeast vacuolar and antioxidant gene deletion mutants to 4I3M, indicating 4I3M negatively affects both cellular ion and “redox” homeostasis in fungi [68]. We previously observed similar results with thymol [58], further indicating 4I3M and thymol share analogous cellular targets in fungi. 4I3M is a synthetic analog of thymol, a natural product. As with thymol, 4I3M has been used as an antimicrobial preservative in personal care products, where 4I3M is more appealing to consumer perception compared to thymol due to its color/odor-neutral characteristic [68].

**Figure 4:**
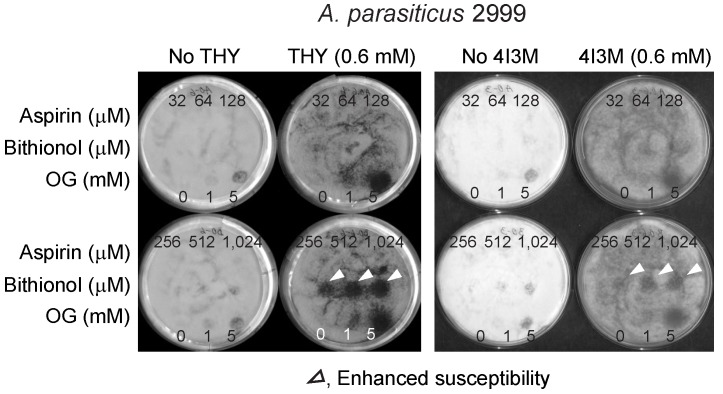
Enhancement of antifungal activity of bithionol by thymol or 4I3M tested in the aflatoxigenic *A. parasiticus* 2999. THY, thymol; 4I3M, 4-isopropyl-3-methylphenol; OG, octyl gallate (positive control).

We applied the same strategy in *Aspergillus parasiticus*, a mycotoxigenic fungus producing hepato-carcinogenic aflatoxins. Chemosensitization effects of thymol or 4I3M to bithionol were also observed in *A. parasiticus* (Figure 4). However, unlike in *A. fumigatus*, thymol exhibited higher sensitizing activity compared to its analog 4I3M in *A. parasiticus* (Figure 4). Also, the sizes of zone of inhibition in *A. parasiticus* were generally smaller than that observed in *A. fumigatus*, indicating “strain-specificity” also exists for the efficacy of chemosensitization when bithionol is co-applied with thymol or 4I3M.

*A. fumigatus* is a human/animal pathogen, while *A. parasiticus* mostly contaminates crops or foods. We recently observed that *A. fumigatus* was able to survive at high temperature (55 °C), while *A. parasiticus* could not grow at the same temperature [69]. Although such differences in niches and/or environmental responsiveness might contribute to the “strain-specificity” determined in this study, elucidation of precise mechanisms exerting strain-specificity warrants future investigation. 

#### 3.2.3. 3,5-Dimethoxybenzaldehyde as a Chemosensitizer to Bithionol or Aspirin

We investigated the effect of other types of chemosensitizer for the enhancement of bithionol activity. The 3,5-dimethoxybenzaldehyde (3,5-D) (Figure 1f) also targets antioxidant systems in fungi, as determined in the model yeast *S. cerevisiae* (Supplementary Table S2 [59,70]). 3,5-D negatively affected the cellular antioxidant system, such as superoxide dismutase or glutathione reductase [59].

**Figure 5:**
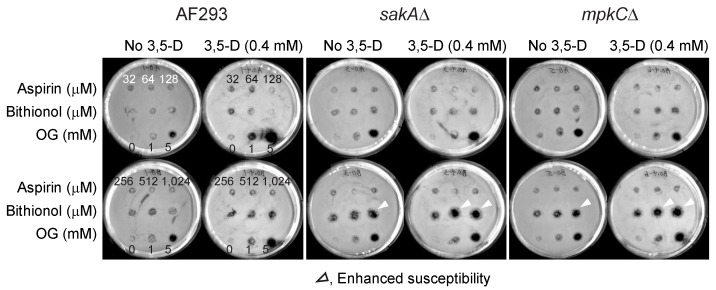
Enhancement of antifungal activity of bithionol by 3,5-dimethoxybenzaldehyde tested in *A. fumigatus*. 3,5-D, 3,5-dimethoxybenzaldehyde; OG, octyl gallate (positive control).

As determined in thymol or 4I3M co-treatment, antifungal activity of bithionol was also enhanced when the drug was co-applied with 3,5-D (Figure 5). Similar to thymol or 4I3M co-treatment, antifungal activity of aspirin was almost unaffected when 3,5-D was co-applied as a chemosensitizer (Figure 5). In general, the level of the enhancement of bithionol activity with 3,5-D was lower than that observed with thymol or 4I3M, which was reflected in smaller sizes of zone of inhibition. 

**Figure 6:**
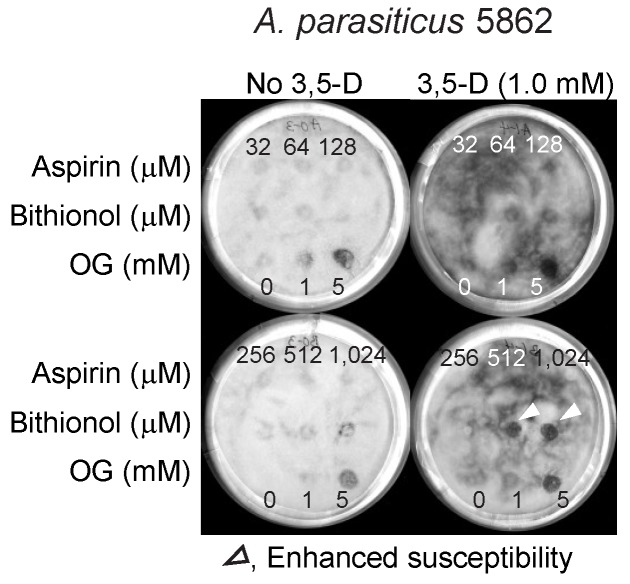
Enhancement of the antifungal activity of bithionol by 3,5-dimethoxybenzaldehyde tested in the aflatoxigenic *A. parasiticus* 5862. 3,5-D, 3,5-dimethoxybenzaldehyde; OG, octyl gallate (positive control).

Similar results were obtained in the aflatoxin-producing *A. parasiticus*, where the sizes of zone of inhibition were generally smaller than that observed in *A. fumigatus*. Therefore, results indicated that “strain-specificity” also existed for the enhancement of the efficacy of “bithionol and 3,5-dimethoxybenzaldehyde” co-treatment in different *Aspergillus* species (Figure 6).

We also speculate that quantitation of the precise level of interactions between compounds—such as synergistic, additive, neutral or antagonistic interactions—during chemosensitization can be determined in future investigations by the methods outlined by the Clinical Laboratory Standards Institute (CLSI) M38-A [71] or the European Committee on Antimicrobial Susceptibility Testing (EUCAST) [72]; definitive document EDef 7.2.].

### 3.3. Scheme of High-Efficiency Drug Repurposing Design 

**Figure 7:**
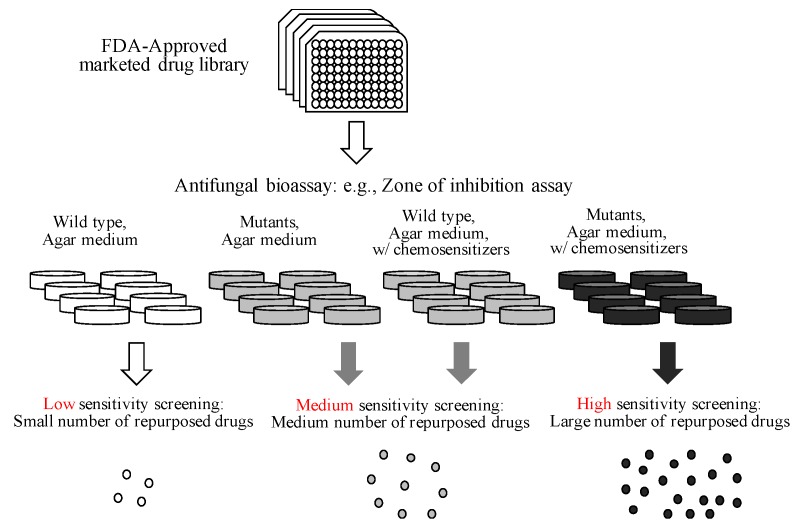
Scheme of high-efficiency drug repurposing design.

Figure 7 describes the scheme of high-efficiency drug repurposing design based on the current investigation, which targets the fungal antioxidant system. When the wild type strain is used during the drug repurposing process, it is considered a “low sensitivity” screening, and thus we expect to obtain a small number of repurposed drugs. When mutants, such as antioxidant mutants, are used or the wild type is used with redox-active sensitizing agents, it is considered a “medium sensitivity” screening, and thus we expect to obtain a medium number of repurposed drugs. When antioxidant mutants are used with redox-active chemosensitizers, it is considered a “high sensitivity” screening, so that we expect the isolation of a large number of repurposed drugs.

Regarding the chemosensitizers, we speculated that (as determined in this study with thymol/4I3M and bithionol) application of chemosensitizers with similar modes of action or cellular targets as the candidate drugs (e.g., pro-oxidant + pro-oxidant, cell wall/membrane disruptors + cell wall/membrane disruptors, etc.) would result in higher sensitivity of the drug repurposing process. Examples of antifungal chemosensitizers identified include: (a) 4-methoxy-2,3,6-trimethylbenzensulfonyl-substituted *d*-octapeptide sensitizing *Candida* strains to fluconazole [73]; (b) 7-chlorotetrazolo [5,1-c]benzo [1,2,4]triazine sensitizing *Candida* and *Saccharomyces* strains to azole drugs [74]; and (c) benzhydroxamic acid sensitizing *Rhizopus oryzae* to triazoles, such as posaconazole and itraconazole [75] (see also Supplementary Table S2 [59,69,70,76]). Identification of comprehensive numbers of chemosensitizers affecting different cellular targets also requires future in-depth study. 

### 3.4. Overcoming Fludioxonil Tolerance of Aspergillus Fumigatus MAPK Mutants

**Figure 8:**
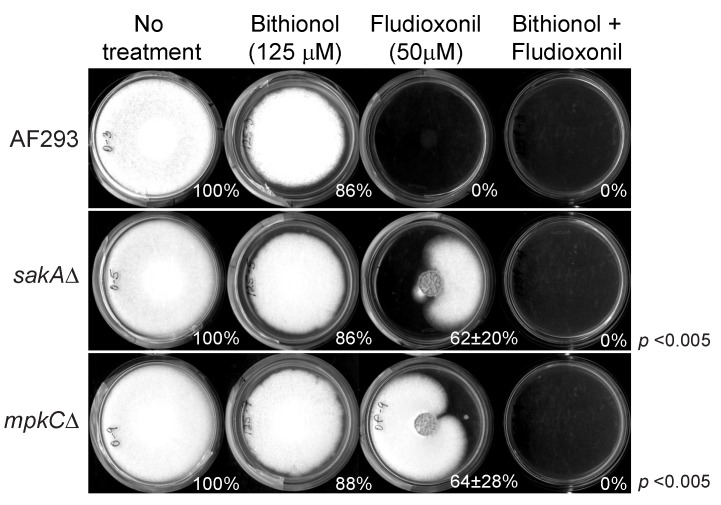
Bithionol overcomes fludioxonil resistance of *Aspergillus fumigatus* MAPK mutants.

Fludioxonil (phenylpyrrole) is a commercial antifungal agent, which induces abnormal and excessive stimulation of the oxidative stress MAPK signaling system [77]. However, fungi having mutations in oxidative stress MAPK pathway can escape the fludioxonil toxicity [77]. As shown in Figure 8, two MAPK mutants (*sakA*Δ, *mpkC*Δ) of *A. fumigatus* exhibited tolerance to fludioxonil at 50 μM, and developed radial growth on PDA, while the growth of the wild type was completely disrupted. However, co-application of sub-fungicidal concentration of bithionol with fludioxonil effectively prevented fungal tolerance to fludioxonil, therefore achieving complete inhibition of the growth of two MAPK mutants. The Student’s *t*-test for paired data (combined, i.e., bithionol + fludioxonil) was versus bithionol alone or fludioxonil alone and determined in *sakA*Δ and *mpkC*Δ strains, where the *p* values for “combined” were determined as <0.005 for both versus bithionol only and fludioxonil only.

In fungi, environmental signals, such as oxidative stress signals, are integrated into the MAPK signaling system, regulating the expression of downstream genes that are countering the stress [78,79]. Therefore, we speculate that, by co-applying a pro-oxidant agent (such as bithionol) with fludioxonil, these tolerant MAPK mutants became more susceptible to the treatment, since the mutated MAPK system of fungi was incapable of initiating a fully operational oxidative stress response including the production of antioxidant enzymes. 

## 4. Conclusions

In summary, a high sensitivity antifungal screening method was investigated by incorporating redox-active chemosensitizers and antioxidant mutants of *A. fumigatus*. Redox-active compounds, such as thymol, 4I3M or 3,5-D, can be used as potent chemosensitizers to enhance antimycotic activity of the repurposed drug bithionol, while the efficacy of the other drug aspirin was almost not affected, indicating “chemosensitizer–drug specificity” exists. The difference in antifungal efficacy between bithionol and aspirin could be based on their role as either “pro-oxidant (bithionol)” or “antioxidant (aspirin)” during treatments (see above). While similar enhancement of antifungal efficacy was also observed in the mycotoxin-producing *A. parasiticus*, the level of sensitivity of this species to the treatments was not comparable to that of *A. fumigatus*, thus indicating “strain-specificity” also exists during chemosensitization. Application of compounds with similar mechanisms of action (e.g., pro-oxidant, cell wall/membrane disruptors, etc.) or cellular targets (e.g., antioxidant system, cell wall/membrane integrity system, etc.) to that of the candidate drugs is suggested, which would result in higher sensitivity of the drug repurposing process.

Current results could be used for the development of high-efficiency, large-scale repositioning of marketed drugs, which can reduce costs, abate resistance, and alleviate negative side effects associated with current antifungal treatments. Inclusion of additional databases, such as DrugCentral [80] and Aggregate Analysis of ClinicalTrials.gov (AACT) [81], etc., might enhance the comprehensiveness of antifungal drug repositioning in the future study.

## Figures and Tables

**Table 1:** Repositioning of non-antifungal drugs to antifungals.

Compounds	Functions	Repositioning Methods	Target Fungi	References
Bithionol	Anti-parasitic drug	High-throughput ATP content assays	*Exserohilum rostratum*	[39]
Tacrolimus	Immunosuppressive agent	The same as above	*E. rostratum*	[39]
Floxuridine	Antimetabolite	The same as above	*E. rostratum*	[39]
Auranofin	Rheumatoid arthritis drug	Clinical & Laboratory Standard Insitute (CLSI) M27-A3 protocol	*Candida* and *Cryptococcus* strains	[40]
Auranofin	Rheumatoid arthritis drug	CLSI M27-A3 (for yeast) & M38-A2 (for filamentous fungi) protocols	*Aspergillus fumigatus*, *Scedosporium apiospermum*, *Lomentospora prolificans*, *Candida albicans*, *Candida krusei*, *Cryptococcus neoformans*	[41]
Drospirenone	Synthetic hormone (birth control pills) w/ethinylestradiol	Enhancement of amphotericin B/caspofungin activity against *Candida albicans* biofilms (96-well plate assay)	*C. albicans*, *Candida glabrata*	[42]
Perhexiline	Anti-anginal agent	The same as above	The same as above	[42]
Toremifine	Selective oestrogen receptor modulator (Oestrogen receptor-positive breast cancer treatment)	The same as above	The same as above	[42]
Aspirin (Acetyl salicylic acid)	Anti-pain, fever, or inflammation drug	European Committee on Antimicrobial Susceptibility Testing (EUCAST) protocol	*C. neoformans*, *Cryptococcus gatti*	[43]
Ibuprofen	Nonsteroidal anti-inflammatory drug	The same as above	The same as above	[43]
Human glycogen synthase kinase 3 (GSK-3) inhibitors	Neurological disorder drug	24-well plate assay	*A. fumigatus*	[44]
Octodrine from Johns Hopkins Clinical Compound Library version 1.0.	Decongestant drug	CLSI M44-A2 protocol	*C. albicans*	[45]
Amiodarone from Prestwick library (Off-patent, biologically active molecules)	Antiarrhythmic drug	High-throughput adenylate kinase assay	*C. neoformans*	[46]
Thioridazine	Antipsychotic drug	The same as above	*C. neoformans*	[46]
Artesunate from Pharmakon 1600 repositioning library	Antimalarial drug	Miconazole synergy test (Anti-biofilm testing)	*C. albicans*	[47]
Hexachlorophene	Anti-infective (topical) drug	The same as above	*C. albicans*	[47]
Pyrvinium pamoate	Antihelmintic drug	The same as above	*C. albicans*	[47]
Quinacrine	Anti-protozoan drug	96-well plate anti-biofilm testing	*C. albicans*	[48]
Cyclo-Phosphamide (plus 28 drugs)	Anti-cancer drug	96-well anti-filamentation assay	*C. albicans*	[49]
Tosedostat from the Enzo & the Institute for Molecular Medicine Finland oncology collection libraries	Anti-cancer (Aminopeptidase inhibitor) drug	EUCAST protocol.	*C. albicans*, *C. glabrata*	[14]
Chloroquine	Anti-malarial drug	Microtiter well plate yeast-to-hyphae transition assay	*C. albicans*	[50]
Aliskiren	Anti-hypertensive drug	CLSI M27-A2 protocol	*C. albicans*	[51]
Atorvastatin	Anti-hypercholestero-laemia drug	CLSI M27-A3 protocol	*C. gatti*	[52]
P21-activated protein kinase inhibitor	Anti-thyroid cancer drug	Agar plate bioassay	*Fusarium oxysporum*, *Fusarium graminearium*, *Phytopthora* sp., *Myrothecium roridum*, *Helminthosporium maydis.*	[53]

## Supplementary Materials

The following are available online at https://www.mdpi.com/2409-9279/2/2/31/s1, Table S1: Pharmacology of repositioned compounds, Table S2: Examples of chemosensitizers targeting antioxidant or cell wall systems in fungi previously determined in the model yeast *Saccharomyces cerevisiae*.
