# Supplementary material for: High Efficiency Drug Repurposing Design for New Antifungal Agents
Source: Methods Protoc. 2019 Apr 17;2(2):31. doi: 10.3390/mps2020031 (PMC6632159; doi:10.3390/mps2020031)
Supplement: Supplementary file 1 [file mps-02-00031-s001.pdf]

SUPPLEMENTARY TABLES

Table S1. Pharmacology of repositioned compounds.

Compounds	Pharmacology
Bithionol [18] 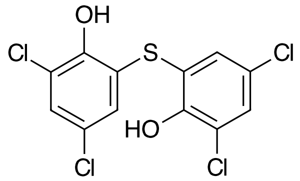	• Halogenated anti-infective agent that is used against trematode and cestode infestations. • Inhibits human soluble adenylyl cyclase
Tacrolimus [19] 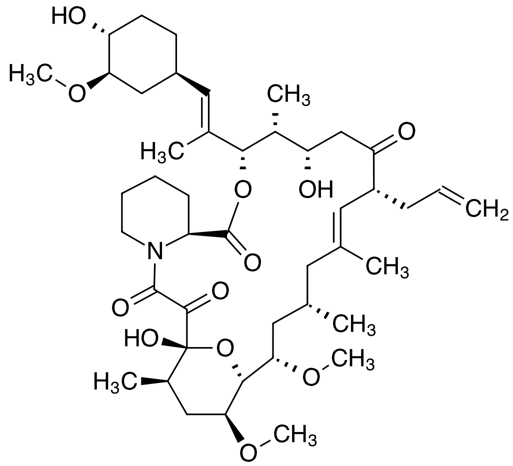	• Inhibits calcineurin phosphatase activity, thus decreases cytokine production. • Exhibits immunosuppressive activity (more potent than cyclosporine). • Prevents T-lymphocyte activation in response to antigenic or mitogenic stimulation.
Floxuridine [20] 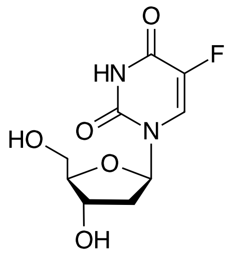	• Fluorinated pyrimidine monophosphate analog of 5-fluoro-2'-deoxyuridine-5'-phosphate (FUDR-MP) with antineoplastic activity. • Inhibits thymidylate synthase, thus disrupting DNA synthesis. • Fluorouracil, the metabolized product, incorporates into RNA, which prevents the utilization of uracil in RNA synthesis.
Auranofin [21] 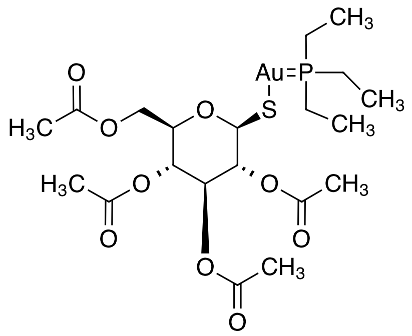	• Inhibits the activity of mitochondrial thioredoxin reductase (TrxR) by interacting with selenocysteine within the redox-active domain. • Induces mitochondrial oxidative stress, thus resulting in the induction of apoptosis. • Inhibits the JAK1/STAT3 signaling pathway, hence suppresses the expression of immune factors involved in inflammation.

Drospirenone [22]<br>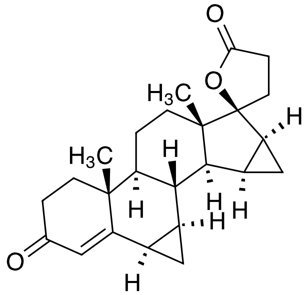	• Possesses progestational and anti-mineralocorticoid activity.<br>• Binds to the progesterone receptor, resulting in a suppression/inhibition of LH activity and ovulation (oral contraceptive).
Perhexiline [23]<br>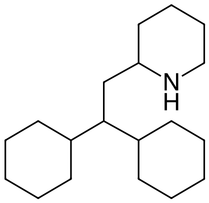	• Binds to the mitochondrial enzyme carnitine palmitoyltransferase (CPT)-1 and CPT-2.<br>• Shifts myocardial substrate utilization, viz., shifting from long chain fatty acids to carbohydrates via the inhibition of CPT-1 and CPT-2, thus used for the therapy in patients with ischaemia.<br>• May cause neuropathy and hepatitis.
Toremifene [24]<br>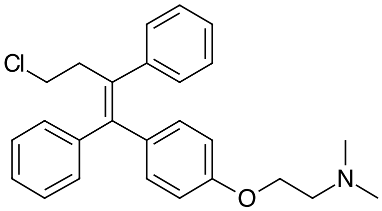	• Selectively modulates estrogen receptors by competitively binds to the receptors, thus interferes with estrogen activity.
Aspirin (Acetylsalicylic acid) [25]<br>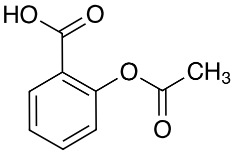	• Non-steroidal anti-inflammatory agent.<br>• Binds to/acetylates serine residues in cyclooxygenases.<br>• Decreased synthesis of prostaglandin, platelet aggregation, and inflammation.
Ibuprofen [26]<br>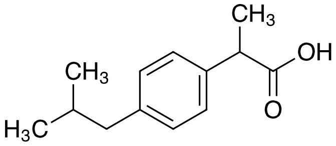	• An anti-inflammatory drug possessing anti-inflammatory, analgesic, and antipyretic activities.<br>• Inhibits cyclo-oxygenase I and II, thus results in a decreased prostaglandin synthesis.
Human glycogen synthase kinase 3 (GSK-3) inhibitors:<br>Tideglusib,<br>TDZD-8,<br>DIP-3.18	• Not available

Octodrine [27] 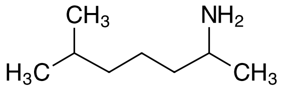	• Small molecule agonist of the estrogen related receptor (ERR) signaling pathway from Tox21 library screening.
Amiodarone [28] 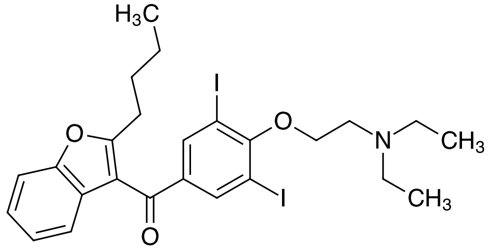	• A class III antiarrhythmic agent possessing antiarrhythmic and vasodilatory activities.<br>• Blocks the myocardial calcium, potassium and sodium channels, thus resulting in prolongation of the cardiac action potential and refractory period.
Thioridazine [29] 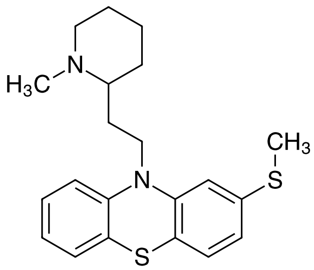	• Binds to dopamine receptors, thus blocking the actions of dopamine or other agonists.
Artesunate [30] 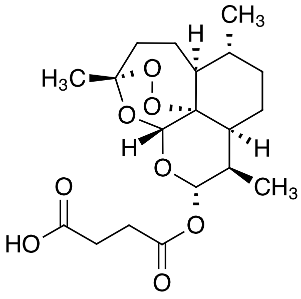	• Anti-malarial, anti-shistosomiasis, antiviral, and potential anti-neoplastic drug.<br>• Forms reactive oxygen species (ROS) and carbon-centered radicals, which damage parasitic organisms.<br>• Induces DNA breakage, arrests the cell cycle (G1 and G2/M phases), inhibits cell proliferation, and induces apoptosis via mitochondrial and caspase signal transduction pathways.
Hexachlorophene [31] 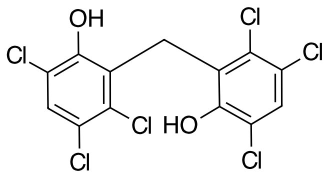	• A chlorinated bisphenol antiseptic agent with bacteriostatic activity.<br>• Active against gram-positive organisms, (Less effective against gram-negative bacteria).
Pyrvinium pamoate [32]	• Anti-parasitic agent (Used therapeutically for treating helminthiasis)

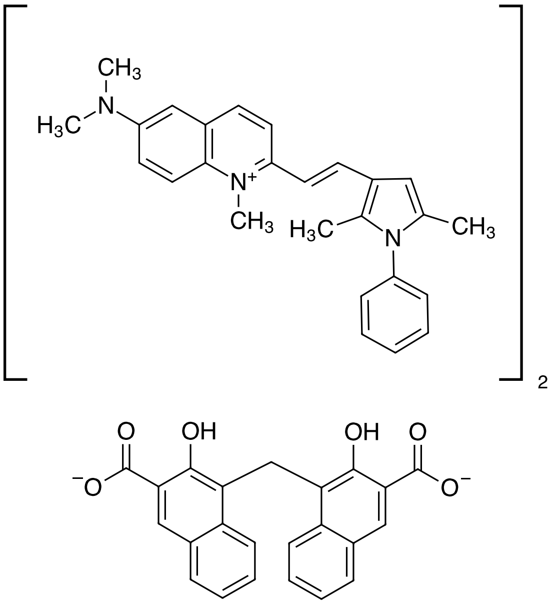	
Quinacrine [33]<br>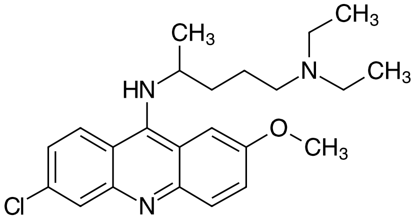	• Potential antineoplastic and antiparasitic agent.<br>• Inhibits the transcription/activity of nuclear factor-kappaB (NF-kappaB), thus inducing/restoring the expression of tumor suppressor p53, p53-dependent apoptotic pathways, tumor cell apoptosis, etc.
Cyclophosphamide [34]<br>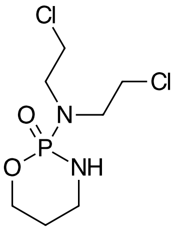	• An alkylating agent possessing antineoplastic and immunosuppressive activities.<br>• The metabolized product phosphoramide mustard binds to and crosslinks DNA and RNA, thus resulting in the inhibition of DNA replication and protein synthesis.
Tosedostat [35]<br>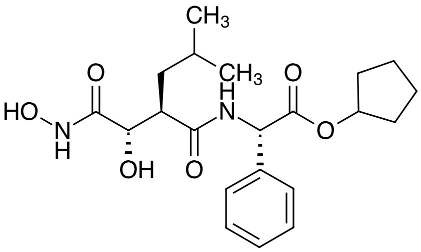	• An inhibitor of aminopeptidases (M1 family) possessing antineoplastic activity.<br>• Inhibits protein synthesis and enhances cancer cell death via the increase of Noxa (proapototic protein) level.
Chloroquine [36]	• An antimalarial, anti-inflammatory drug.<br>• Inhibits heme polymerase in parasites, thus accumulating toxic heme within the parasitic cell.

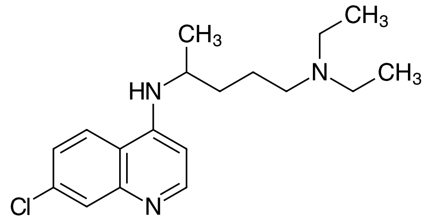	• May interfere with nucleic acid biosynthesis.<br>• Possesses chemosensitizing and radiosensitizing activities in cancer cell.
Aliskiren [37]<br>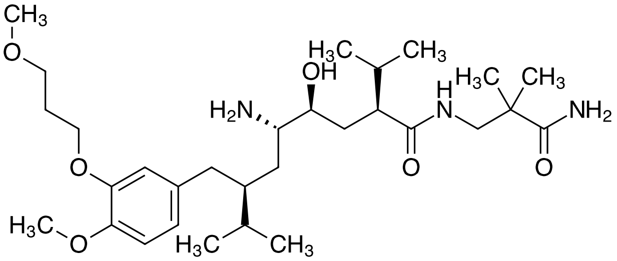	• Non-peptide renin inhibitor with antihypertensive activity.<br>• Selectively binds to the S3 sub-pocket of renin, thus preventing arterial vasoconstriction and inhibiting aldosterone production.
Atorvastatin [38]<br>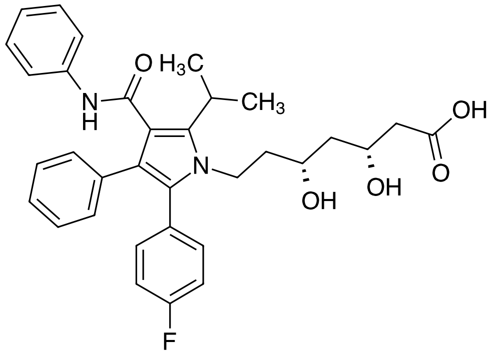	• A lipid-lowering agent.<br>• Competitively inhibits hepatic hydroxymethyl-glutaryl coenzyme (HMG-CoA) reductase, thus lowers the levels of plasma cholesterol and lipoprotein levels.<br>• Modulates immune responses.
P21-activated protein kinase inhibitor	• Not available

Table S2. Examples of chemosensitizers targeting antioxidant or cell wall systems in fungi previously determined in the model yeast *Saccharomyces cerevisiae*. [1]

**Compounds**	**Antioxidant targets[5]**	**Cell wall targets[5]**	**References**
Octyl gallate	*yap1*Δ	*slt2*Δ, *bck1*Δ	[69]
trans-Cinnamaldehyde	*sod1*Δ, *sod2*Δ	ND[2]	[59]
2-Hydroxy-5-methoxy-benzaldehyde	*sod1*Δ, *sod2*Δ	ND[2]	[59]
2-Hydroxy-4-methoxy-benzaldehyde	ND[2]	*slt2*Δ, *bck1*Δ	[70]
3,5-Dimethoxy-benzaldehyde	*sod1*Δ, *sod2*Δ, *glr1*Δ	*slt2*Δ, *bck1*Δ	[59,70]
2,5-Dimethoxy-benzaldehyde	*sod1*Δ, *sod2*Δ	*slt2*Δ, *bck1*Δ	[59,70]
2,3-Dihydroxy-benzaldehyde	*sod1*Δ, *sod2*Δ, *glr1*Δ	ND[2]	[76]
4-Methoxybenzoic acid	ND[2]	*slt2*Δ, *bck1*Δ	[70]

[1]Functions of gene products: Sod1, Cytosolic superoxide dismutase; Sod2, Mitochondrial superoxide dismutase; Glr1, Glutathione reductase; Slt2, MAPK of cell wall integrity system; Bck1, MAPKKK of cell wall integrity system.
[2]Not determined
